# Migraine treatment and healthcare costs: retrospective analysis of the China Health Insurance Research Association (CHIRA) database

**DOI:** 10.1186/s10194-020-01117-2

**Published:** 2020-05-13

**Authors:** Shengyuan Yu, Yanlei Zhang, Yuan Yao, Haijun Cao

**Affiliations:** 1 Department of Neurology, Chinese PLA General Hospital, Beijing, China; 2 Lilly Suzhou Pharmaceutical Co., Ltd. Shanghai Branch, 19F, Centre T1, HKRI Taikoo, No. 288, Shimen No.1 Road, Shanghai, 200021 China

**Keywords:** Migraine disorder, Adult, Drug prescriptions, Acute medication, Preventive medication, Retrospective studies

## Abstract

**Background:**

Adult migraine remains underdiagnosed and undertreated, despite significant negative effects on physical and emotional functioning. Information on prescribing patterns and treatment costs of migraine in China is limited.

**Methods:**

This retrospective analysis of the China Health Insurance Research Association (CHIRA) medical insurance claims database in 2016 to 2017 evaluated treatment patterns, direct medical costs, and healthcare resource utilization among adults with migraine in mainland China.

**Results:**

Of 108,375 patients with headache-related outpatient visits, 10,652 were adults with migraine (mean age 51.4 years, 55.4% female). Common comorbidities were major depressive disorder (4.1%), insomnia (3.8%), and anxiety (2.3%). Migraine patients were prescribed acute medication (26.4%), preventive medication (15.0%), and Chinese patent and herbal medicines (24.5% and 11.7%, respectively). Of patients prescribed acute medication, 68.8% received non-aspirin non-steroidal anti-inflammatory drugs (NSAIDs), 7.1% received opioids, while only 3.3% received triptans. Mean annual outpatient costs per patient were 46.5 United States dollars (USD), with mean (standard deviation) 1.8 (2.0) outpatient visits per year. Medication costs for traditional Chinese medicine (22.4 USD per patient) were higher than for Western medicine (13.5 USD).

**Conclusion:**

Among migraine patients in China, NSAIDs were commonly prescribed as acute medication, while utilization of migraine-specific triptans and preventive medications was low.

## Background

Migraine is a primary headache disorder characterized by disabling attacks of moderate-to-severe pain, accompanied by nausea, photophobia, or phonophobia, which impair ability to function and adversely impact quality of life [1–3]. Migraine has an estimated global prevalence of 14.4% and is the second highest cause of years lived with disability based on the Global Burden of Disease Study [3].

In China, population-based estimates of the 1-year prevalence of migraine range from 7.9% to 14.3% in adults [4–6]. Health-related quality-of-life studies in China, including Taiwan, have demonstrated substantial negative effects of migraine on physical and emotional functioning, impacting work, household, and leisure activities [5, 7–9]. In a population of 1.4 billion, migraine presents a significant clinical burden in China.

Migraine treatment guidelines for China are consistent with those for Europe and the United States (US) in supporting a stratified approach to acute (abortive) treatment, whereby medication choice is based on attack severity and symptoms, the efficacy and side effect profile of the drug, and the patient’s previous response to acute treatment [10–12]. Chinese guidelines recommend non-steroidal anti-inflammatory drugs (NSAIDs), acetaminophen, compound analgesics containing caffeine (cautioning that caffeine could increase the risk of drug addiction and medication overuse headache), and triptans; opioids, barbiturates, and ergotamine derivatives are not recommended for regular use [10].

Preventive (prophylaxis) treatment can be offered in the case of recurring migraine that is interfering with daily routine despite acute treatment; failure of, contraindication to, or side effects from acute medications; risk of acute medication overuse; circumstances such as hemiplegic migraine; high or increasing attack frequency; and patient preference [13]. Metoprolol, propranolol, flunarizine, valproic acid, and topiramate are the recommended preventives based on Level A evidence in Chinese and European guidelines [10, 11]. An estimated 34% to 39% of patients with migraine in Europe and the US are eligible for preventive migraine treatment [14, 15]. However, in the European Eurolight study, less than 14% of eligible patients actually received preventive medication, and in the American Migraine Prevalence and Prevention (AMPP) study, 32% of never-users of preventive treatment would benefit from their use, suggesting undertreatment and inadequate disease management [14, 15].

Information on the prescribing patterns and treatment costs of migraine in mainland China is limited. Although there is substantial evidence for the economic burden of migraine in the US and Europe, this information cannot be generalized to China because of the differences in diagnosis and treatment practices, availability of prescription medication, and use of traditional Chinese medicine alongside Western medicine. A population-based survey in China in 2009 estimated the total annual direct costs of migraine diagnosis and treatment to be 58.0 billion Chinese yuan renminbi (CNY) (8.4 billion US dollars [USD]) [5]. However, this survey relied on patient-reported information for migraine diagnosis and healthcare resource use. Hence, there is a need for China-specific data reflecting local diagnostic and treatment practices and the use of complementary medicine.

This retrospective analysis of the China Health Insurance Research Association (CHIRA) nationwide medical insurance claims data aimed to understand the treatment patterns, direct medical costs, and healthcare resource utilization among adult patients with migraine in mainland China.

## Methods

### Dataset

The CHIRA database is a medical insurance management information system initiated in 2007 that contains nationwide consecutive inpatient and outpatient visit claims data of urban basic medical insurance in China. With approximately 95% of the population in mainland China covered by public medical insurance, the CHIRA database is a valuable resource of real-world evidence for medical costs in China [16]. CHIRA data are collected annually from the local insurance centers of a random de-identified sample of selected areas of China including at least 2% of municipalities, at least 2% of provincial capital cities, and at least 5% of prefecture-level cities. The database contains patient basic information, medical institution information, diagnosis results, healthcare service utilization, medication prescription, and healthcare expenditure details.

CHIRA granted permission to access the anonymized database for the purposes of this study. Only anonymized information was accessible from the CHIRA database and therefore institutional ethics approval and informed consent were not required.

### Patient selection

All outpatient visit and healthcare use information for the diagnosis and treatment of headache in 2016 and 2017 was extracted from the CHIRA database. Adult patients (≥18 years) were selected who had a primary diagnosis of migraine, identified by *International Statistical Classification of Diseases and Related Health Problems revision 10* (*ICD-10*) code (*ICD-10* G43.0, G43.1, G43.2, G43.3, G43.8, G43.9) and supplemented by searching physician Chinese character descriptions against translations from the World Health Organization Chinese version of *ICD-10*. Patients were excluded if they had cluster headache (*ICD-10* G44.0), malignancy, schizophrenia, or stroke, or were receiving hemodialysis or peritoneal dialysis.

### Outcome measures

Patient information identified from the CHIRA database included age, gender, comorbidities (predefined as anxiety, major depressive disorder, epilepsy, fremitus, and insomnia), and medical insurance type (Urban Employee Basic Medical Insurance or Urban Residents Basic Medical Insurance).

Medications prescribed in the 2016–2017 period for the treatment of migraine or for pain relief were identified and categorized according to class of acute medication (including aspirin, paracetamol, non-aspirin NSAIDs, opioids, triptans, ergot alkaloids, barbiturates, antiemetics, glucocorticoids, and mannitol) and preventive medication (calcium antagonists, β1-receptor blockers, antiepileptics, antidepressants, and type A botulinum toxin). Medications within each acute and preventive medication category are listed in Additional Table 1. Prescriptions for Chinese patent and herbal medicines were identified using Chinese character search terms. Chinese patent medicines are manufactured products with a standardized composition of herbal extracts and other ingredients, generally available as pills, capsules, or liquids. Chinese patent medicines used in the treatment of migraine generally contain extracts such as *Ligusticum wallichii*, *Angelica dahurica* root, and *Gastrodia elata*, with the aim of promoting circulation and relieving pain. Chinese herbal medicines are herbal decoctions, often condensed into granules or powder.

Healthcare resource utilization included the hospital level and department of the patient’s first migraine-related visit in the 2016–2017 period. Diagnostic methods were identified using Chinese character keyword searches for cranial computed tomography (CT), cranial magnetic resonance imaging (MRI), transcranial Doppler ultrasonography, and electroencephalography (EEG).

Annual visit frequency and direct medical costs were calculated in the subgroup of patients with a migraine-related outpatient visit in January of 2016 or 2017 and with at least 11 months of follow-up. Medication costs were calculated for prescribed Western medicine and for Chinese patent or herbal medicine. Diagnostic costs included claims for cranial CT, cranial MRI, transcranial Doppler ultrasonography, and EEG. Non-medication costs included physical therapy and oxygen treatment.

### Statistical analysis

Study variables were summarized using descriptive statistics. Analyses were performed using STATA/SE 14.0 (StataCorp) software (College Station, TX: StataCorp LP).

## Results

### Patient population and characteristics

**Fig. 1:**
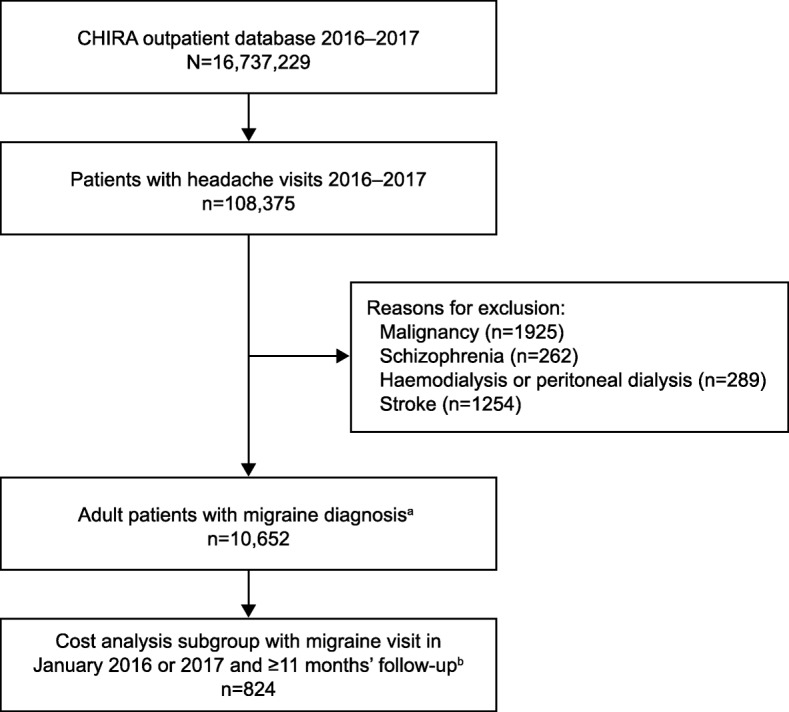
Patient selection. ^a^Migraine diagnosis by *ICD-10* code (*ICD-10* G43.0, G43.1, G43.2, G43.3, G43.8, G43.9) supplemented by physician Chinese characters descriptions. ^b^Patients with a migraine-related outpatient visit in January 2016 or January 2017 and with ≥11 months of follow-up were included in the outpatient cost analysis. CHIRA: China Health Insurance Research Association; *ICD*: *International Statistical Classification of Diseases and Related Health Problems*

Data from over 16 million patients were retrieved from the CHIRA outpatient database in the calendar years of 2016 and 2017. A total of 108,375 patients had headache-related outpatient visits, of whom 10,652 adult patients with migraine met the eligibility criteria and were included in the analysis (Fig. 1).

Mean age was 51.4 years (standard deviation [SD] 15.8 years) and 55.4% of patients were female (Table 1). Comorbidities included major depressive disorder (4.1%), insomnia (3.8%), and anxiety (2.3%). Of patients for whom first migraine-related visit site was recorded, over two-thirds visited a primary-level hospital; 19.2% visited a tertiary-level hospital, most commonly to a neurology department.

**Table 1:** Patient characteristics

	Adults with migraine (*N* = 10,652)
Age, years, mean (SD)	51.4 (15.8)
Age category, years, n (%)	
18–29	958 (9.0)
30–39	1736 (16.3)
40–49	2222 (20.9)
50–59	2243 (21.1)
≥60	3493 (32.8)
Female, n (%)	5902 (55.4)
Insurance type, n (%)	
UEBMI	7675 (72.1)
URBMI	2977 (27.9)
Comorbidities^a^, n (%)	
Major depressive disorder	442 (4.1)
Insomnia	402 (3.8)
Anxiety	248 (2.3)
Epilepsy	46 (0.4)
Fremitus	17 (0.2)
Hospital level of first visit^b^, n (%)	(*n* = 9982)
Primary	6686 (67.0)
Secondary	1384 (13.9)
Tertiary	1912 (19.2)
Department of tertiary hospital first visit^b^, n (%)	(*n* = 979)
Neurology	402 (41.1)
Traditional Chinese medicine	130 (13.3)
Internal medicine	128 (13.1)
Emergency room	68 (6.9)
Other	251 (25.6)

*CHIRA* China Health Insurance Research Association, *SD* standard deviation, *UEBMI* Urban Employee Basic Medical Insurance, *URBMI* Urban Residents Basic Medical Insurance

^a^Incidence of predefined comorbidities recorded in the period 2016–2017 in the CHIRA database

^b^First migraine-related visit in the period 2016–2017 in the CHIRA database

### Prescribed migraine medication

Acute medication was prescribed for 2813 (26.4%) patients (Table 2). The majority (68.8%) of patients receiving acute medication were prescribed non-aspirin NSAIDs, with ibuprofen being the most common (36.5%). Other classes of acute medication were each prescribed to ≤8.0% of patients receiving acute medication, including opioids (7.1%) and ergot alkaloids (6.1%). Only 3.3% of patients receiving acute medication were prescribed a triptan.

**Table 2:** Acute medication prescribed for migraine

	Adults with migraine (*N* = 10,652)
Patients prescribed ≥1 acute medication, n (%)	2813 (26.4)
Types of acute medication, n (%)	(*n* = 2813)
Non-aspirin NSAID	1934 (68.8)
Aspirin	225 (8.0)
Weak opioids/opioids	199 (7.1)
Ergot alkaloids	172 (6.1)
Acetaminophen	122 (4.3)
Triptans	92 (3.3)
Antiemetics	53 (1.9)
Others	199 (7.1)

*NSAID* non-steroidal anti-inflammatory drug

Preventive medication was prescribed for 1602 (15.0%) patients (Table 3). Of patients receiving preventive medication, the majority (88.3%) were prescribed calcium antagonists, primarily flunarizine (87.6%), and 8.4% were prescribed β1-receptor antagonists. Other classes were each prescribed to less than 4% of the patients receiving preventive medication.

**Table 3:** Preventive medication prescribed for migraine

	Adults with migraine (*N* = 10,652)
Patients prescribed ≥1 preventive medication, n (%)	1602 (15.0)
Types of preventive medication, n (%)	(*n* = 1602)
Calcium antagonists	1414 (88.3)
β1-receptor antagonists	135 (8.4)
Antiepileptics	43 (2.7)
Antidepressants	25 (1.6)
Type A botulinum toxin	1 (0.1)
Others	54 (3.4)

Chinese patent medicine was prescribed for 2612 (24.5%) patients and Chinese herbal medicine was prescribed for 1248 (11.7%) patients. The proportions of patients prescribed Chinese patent or herbal medicine were comparable with those prescribed Western acute (26.4%) or preventive (15.0%) medications. Traditional Chinese medicines were prescribed at 64.3% of all migraine-related outpatient visits.

### Healthcare resource utilization and costs

Mean annual number of outpatient visits per patient was 1.8 (SD 2.0; median, 1.0). Healthcare resources for migraine diagnosis included cranial CT (4.2% of visits), cranial MRI (1.8%), transcranial Doppler ultrasonography (1.2%), and EEG (0.1%).

The cost analysis subgroup included 824 patients with a migraine-related outpatient visit in January of 2016 or 2017 and with at least 11 months of follow-up (Fig. 1). Mean annual outpatient costs per patient were 46.5 USD, with medication costs accounting for 36.0 USD and diagnostic and non-medication costs accounting for 10.5 USD (Table 4). Medication costs for traditional Chinese medicine (22.4 USD per patient) were higher than costs for Western medicine (13.5 USD).

**Table 4:** Annual outpatient costs per patient

N=824^a^	Costs, USD^b^	
	Mean (SD)	Median
Total annual costs per patient	46.5 (80.8)	20.6
Medication costs	36.0 (73.7)	15.9
Western medicine	13.5 (23.4)	6.5
Traditional Chinese medicine	22.4 (43.3)	7.6
Diagnostic and non-medication costs^c^	10.5 (30.7)	0.9

*USD* United States dollars

^a^Patients with a migraine-related outpatient visit in January of 2016 or 2017 and with ≥11 months of follow-up

^b^Exchange rate: 1 USD = 6.697 Chinese yuan renminbi

^c^Migraine diagnostic costs included cranial computed tomography, cranial magnetic resonance imaging, transcranial Doppler ultrasonography, and electroencephalography; migraine non-medication costs included physical therapy and oxygen treatment

## Discussion

This is the first study to describe the treatment patterns and costs of migraine treatment in urban mainland China using a retrospective analysis of the large CHIRA medical insurance claims database. The treatment patterns for adult migraine patients in this study indicate relatively low levels of headache management in China. Only 26.4% of patients were prescribed acute medication, which contrasts with higher rates (73%) from Japanese insurance claims data [17]; this may reflect cultural differences in prescribing patterns or greater reliance on over-the-counter rather than prescription acute medications in China. In the US, approximately half of patients used prescription acute medication, with or without over-the-counter acute medication [18], and much higher rates were reported among patients consulting specialist neurologists and physicians for their migraine [19].

Non-aspirin NSAIDs, primarily ibuprofen, were by far the most commonly prescribed acute medication in our study, which is in accordance with international treatment guidelines [10–12] and may also reflect their accessibility and relatively low cost. There is evidence to support ibuprofen as effective for severe migraine attacks; however, NSAIDs are non–migraine-specific, and their acute use can be associated with peptic ulcer or renal disease, which should be considered in treatment decisions [12].

Triptans are a class of recommended migraine-specific acute medication [10–12] and are also effective in about 60% of non-responders to NSAIDs [20]. Three types of triptan are commercially available in China: sumatriptan, zolmitriptan, and rizatriptan. Although the Level A evidence supporting triptan use is recognized in Chinese as well as international treatment guidelines [10–12], the rate of triptan prescribing in this study was low; of patients receiving acute medication, only 3.3% were prescribed triptans. Surveys of migraine patients attending hospital neurology departments in mainland China have also found triptan use to be rare [21, 22]. In contrast, triptans are among the most commonly prescribed acute medications for migraine in Europe, the US, and Japan [17, 19, 23, 24]. The lower rate of triptan use in China may be explained by barriers to access owing to limited triptan availability in most hospitals and pharmacies. Limited over-the-counter purchase of triptans has also been reported in China, with their relatively high price making triptans unaffordable to many patients with migraine [22].

In our study, opioids were prescribed for 7.1% of patients receiving acute medication. In contrast, more patients are prescribed opioids for migraine in the US, from 16% in the AMPP study [25] to 36% in the Chronic Migraine Epidemiology and Outcomes study [26]. The consumption of opioids in China is far lower than the global average, with a mean defined daily opioid dose in 2011 to 2013 of 96 units per million people per day in China compared with 3027 units globally [27]. Opioids are subject to government regulation in China [28] and are not recommended for the acute treatment of migraine in Chinese, US, or European guidelines [10–12] due to the potential for medication overuse and transformation from episodic to chronic migraine [29, 30]. Although we cannot determine whether the 7.1% of patients who were prescribed opioids in our study were non-responders to other medications, this rate of opioid prescription demonstrates an unmet need for additional migraine-specific acute treatments that lack the safety concerns of opioids.

Preventive medication was prescribed for only 15% of patients in our study, consistent with insurance claims data for Japan, which showed that 15% of patients received preventive treatment. However, these rates are well below the estimated 34% to 39% of patients who would benefit from migraine preventive treatment reported for Europe and the US [14, 15]. The limited available evidence from other studies in China also suggests that preventive medications are underutilized, with ≤5% of patients receiving preventive medication [21, 31]. In our study, the calcium-channel blocker flunarizine was the most commonly prescribed preventive, used by 87.6% of patients receiving preventives. The preferential use of flunarizine over other migraine preventives in China may reflect its relatively mild adverse effects, which can include fatigue, weight gain, and mood changes [32].

Approximately one quarter of patients in our study were prescribed Chinese patent medicine and over 11% were prescribed Chinese herbal medicine. Moreover, the mean annual direct costs for traditional Chinese medicine (patent or herbal) were almost twice the estimated direct cost of prescribed Western medicine. Although a meta-analysis has suggested efficacy for Chinese patent medicines in the treatment of migraine, many available studies are of poor methodological quality and the potential mechanisms of action are unclear [33]. This is consistent with the finding by Luo et al. of lower rates of satisfaction with Chinese patent medicine compared with Western medicine in migraine treatment [22]. Given the high direct costs of traditional Chinese medicine shown in our study and potentially low rates of satisfaction, there is a need for greater understanding of their efficacy in migraine treatment and the factors driving the prescription of traditional Chinese medicine in preference to Western medicine.

The mean total annual outpatient costs for medication, diagnostic tests, and non-medication costs for physical therapy or oxygen treatment were estimated to be 46.5 USD per patient. This is lower than the annual direct costs previously reported by Yu et al. for migraine diagnosis and treatment in China, which were estimated to be 105 USD per patient [5]. This difference may reflect study design differences, with Yu et al. including all out-of-pocket expenses for diagnosis and treatment, in contrast with our study, which included medical insurance claims and excluded over-the-counter purchases from pharmacies. A retrospective case-control analysis of the Taiwanese nationwide National Health Insurance Research Database estimated annual total drug costs from outpatient visits to be significantly higher for patients with refractory migraine compared with that for non-migraine control subjects, but the estimated costs were higher than reported in our study owing to the inclusion of costs associated with comorbid conditions [34].

The strengths of the current study include the wide urban population coverage of the CHIRA database. Approximately 95% of China’s population is covered by public health insurance [16], and China’s urban population represents approximately 59% of the total population [35]. CHIRA data also represent a range of localities, including municipalities, provincial capital cities, and prefecture-level cities, and include primary- through to tertiary-level hospitals. However, the findings may not be representative of patients in rural areas or those without urban employee or resident basic medical insurance. Patients who did not seek treatment from a healthcare provider and used only over-the-counter medications would also be excluded from the study. In addition, the database does not include medications purchased outside of hospital pharmacies by the enrolled patients and is therefore likely to underestimate medication use. Only patients with a primary diagnosis of migraine were included in the analysis; however, some medications (eg, calcium antagonists) may have been prescribed for other indications, resulting in an overestimation of certain prescriptions for migraine. Lastly, although it was not possible to determine the incidence of chronic and episodic migraine in this population, the cost analysis was based on actual prescription data and, therefore, would reflect the potentially more frequent use of medication in patients with chronic migraine.

## Conclusions

Our findings from the analysis of this large nationwide database are a representative reflection of migraine treatment and costs in urban mainland China. Prescribing rates for acute medication in China were low compared with the US and Japan. NSAIDs were the most commonly prescribed acute medication, while there was low utilization of migraine-specific triptans and preventive medications. Traditional Chinese medicine was commonly prescribed in clinical practice, and with the costs of traditional Chinese medicine comprising the largest proportion of total drug costs. The findings indicate an economic burden associated with the diagnosis and treatment of migraine in China, and possible unmet needs for effective migraine-specific acute treatment as well as preventive medication.

## Supplementary information

**Additional file 1: Table 1.** Predefined search terms for prescription medication.

## Data Availability

The dataset for the current study is sampled from a database of nationwide claims data of urban basic medical insurance, and is not publicly available. The data analysis report is however available from the authors upon reasonable request.

## Abbreviations

AMPP: American Migraine Prevalence and Prevention
CHIRA: China Health Insurance Research Association
CNY: Chinese yuan renminbi
CT: Computed tomography
EG: Electroencephalography
ICD-10: International Statistical Classification of Diseases and Related Health Problems revision 10
MRI: Magnetic resonance imaging
NSAID: Non-steroidal anti-inflammatory drug
SD: Standard deviation
UEBMI: Urban Employee Basic Medical Insurance
URBMI: Urban Residents Basic Medical Insurance
US: United States
USD: United States dollars

## References

[1] Headache Classification Committee of the International Headache Society (IHS) (2018). The International Classification of Headache Disorders, 3rd edition. Cephalalgia.

[2] Blumenfeld AM, Varon SF, Wilcox TK, Buse DC, Kawata AK, Manack A (2011). Disability, HRQoL and resource use among chronic and episodic migraineurs: results from the international burden of migraine study (IBMS). Cephalalgia.

[3] GBD 2016 Headache Collaborators (2018). Global, regional, and national burden of migraine and tension-type headache, 1990–2016: a systematic analysis for the Global Burden of Disease Study 2016. Lancet Neurol.

[4] Luo N, Qi W, Tong W, Tan F, Zhang Q, He J (2014). Prevalence and burden of headache disorders in two neighboring provinces of China. J Clin Neurosci.

[5] Yu S, Liu R, Zhao G, Yang X, Qiao X, Feng J (2012). The prevalence and burden of primary headaches in China: a population-based door-to-door survey. Headache.

[6] Gu X, Xie Y (2018). Migraine attacks among medical students in Soochow University, Southeast China: a cross-sectional study. J Pain Res.

[7] Wang X, Xing Y, Sun J, Zhou H, Yu H, Zhao Y (2016). Prevalence, associated factors, and impact on quality of life of migraine in a community in Northeast China. J Oral Facial Pain Headache.

[8] Wang SJ, Fuh JL, Lu SR, Juang KD (2001). Quality of life differs among headache diagnoses: analysis of SF-36 survey in 901 headache patients. Pain..

[9] Hung PH, Fuh JL, Wang SJ (2006). Validity, reliability and application of the Taiwan version of the migraine disability assessment questionnaire. J Formos Med Assoc.

[10] Chinese Medical Association Group (2016). Guide to the prevention and treatment of migraine in China [Chinese]. Chin J Pain Med.

[11] Evers S, Afra J, Frese A, Goadsby PJ, Linde M, May A (2009). EFNS guideline on the drug treatment of migraine--revised report of an EFNS task force. Eur J Neurol.

[12] Marmura MJ, Silberstein SD, Schwedt TJ (2015). The acute treatment of migraine in adults: the American headache society evidence assessment of migraine pharmacotherapies. Headache.

[13] Parikh SK, Silberstein SD (2019). Preventive treatment for episodic migraine. Neurol Clin.

[14] Lipton RB, Bigal ME, Diamond M, Freitag F, Reed ML, Stewart WF (2007). Migraine prevalence, disease burden, and the need for preventive therapy. Neurology.

[15] Katsarava Z, Mania M, Lampl C, Herberhold J, Steiner TJ (2018). Poor medical care for people with migraine in Europe - evidence from the Eurolight study. J Headache Pain.

[16] Yang Y, Zhang J, Du F, Montgomery W, Li H, Flynn JA (2014). Real world evidence in mainland China: experience with the use of health care claims data. Value Health.

[17] Meyers JL, Davis KL, Lenz RA, Sakai F, Xue F (2019). Treatment patterns and characteristics of patients with migraine in Japan: a retrospective analysis of health insurance claims data. Cephalalgia.

[18] Diamond S, Bigal ME, Silberstein S, Loder E, Reed M, Lipton RB (2007). Patterns of diagnosis and acute and preventive treatment for migraine in the United States: results from the American Migraine Prevalence and Prevention study. Headache.

[19] Ford JH, Jackson J, Milligan G, Cotton S, Ahl J, Aurora SK (2017). A real-world analysis of migraine: a cross-sectional study of disease burden and treatment patterns. Headache.

[20] Diamond ML, Hettiarachchi J, Hilliard B, Sands G, Nett R (2004). Effectiveness of eletriptan in acute migraine: primary care for Excedrin nonresponders. Headache.

[21] Li X, Zhou J, Tan G, Wang Y, Ran L, Chen L (2012). Diagnosis and treatment status of migraine: a clinic-based study in China. J Neurol Sci.

[22] Luo N, Qi W, Zhuang C, Di W, Lu Y, Huang Z (2014). A satisfaction survey of current medicines used for migraine therapy in China: is Chinese patent medicine effective compared with Western medicine for the acute treatment of migraine?. Pain Med.

[23] Bigal ME, Borucho S, Serrano D, Lipton RB (2009). The acute treatment of episodic and chronic migraine in the USA. Cephalalgia.

[24] Vo P, Paris N, Bilitou A, Valena T, Fang J, Naujoks C (2018). Burden of migraine in Europe using self-reported digital diary data from the migraine buddy© application. Neurol Ther.

[25] Buse DC, Pearlman SH, Reed ML, Serrano D, Ng-Mak DS, Lipton RB (2012). Opioid use and dependence among persons with migraine: results of the AMPP study. Headache.

[26] Schwedt TJ, Lipton RB, Friedman BW, Fanning KM, Reed MI (2019). Demographics, headache characteristics, and other factors associated with opioid use in people with migraine: results from the chronic migraine epidemiology and outcomes study. Headache.

[27] Fang W, Liu T, Gu Z, Li Q, Luo C (2019). Consumption trend and prescription pattern of opioid analgesics in China from 2006 to 2015. Eur J Hosp Pharm.

[28] Gu W (2015). Update on administration of anesthetics and psychoactive drugs for pain management in China. Acta Anaesthesiol Taiwanica.

[29] Trang T, Al-Hasani R, Salvemini D, Salter MW, Gutstein H, Cahill CM (2015). Pain and poppies: the good, the bad, and the ugly of opioid analgesics. J Neurosci.

[30] Bigal ME, Serrano D, Buse D, Scher A, Stewart WF, Lipton RB (2008). Acute migraine medications and evolution from episodic to chronic migraine: a longitudinal population-based study. Headache.

[31] Lu SR, Fuh JL, Chen WT, Juang KD, Wang SJ (2001). Chronic daily headache in Taipei, Taiwan: prevalence, follow-up and outcome predictors. Cephalalgia.

[32] Karsan N, Palethorpe D, Rattanawong W, Marin JC, Bhola R, Goadsby PJ (2018). Flunarizine in migraine-related headache prevention: results from 200 patients treated in the UK. Eur J Neurol.

[33] Xiao Y, Yuan L, Liu Y, Sun X, Cheng J, Wang T (2015). Traditional Chinese patent medicine for prophylactic treatment of migraine: a meta-analysis of randomized, double-blind, placebo-controlled trials. Eur J Neurol.

[34] Tang CH, Chen YC, Ng K, Wang SJ (2013). A retrospective matched case-control study on medical costs of refractory migraine in Taiwan. Headache.

[35] World Bank. World development indicators: urban population (% of total population) in China. Available at: http://data.worldbank.org/indicator/SP.URB.TOTL.IN.ZS. Accessed 12 Aug 2019

