# Supplementary material for: Migraine treatment and healthcare costs: retrospective analysis of the China Health Insurance Research Association (CHIRA) database
Source: J Headache Pain. 2020 May 13;21(1):53. doi: 10.1186/s10194-020-01117-2 (PMC7222520; doi:10.1186/s10194-020-01117-2)
Supplement: Supplementary file 1 — Additional file 1: Table 1. Predefined search terms for prescription medication. [file 10194_2020_1117_MOESM1_ESM.docx]

**Additional Table 1.** Predefined search terms for prescription medication.

| **Acute medication** |
| --- |
| Aspirin |
| Aspirin DL-lysine |
| Paracetamol |
| Non-aspirin NSAID |
| Ibuprofen |
| Naproxen |
| Diclofenac |
| NSAID, acetaminophen, and caffeine compounds |
| Weak opioids/opioids |
| Ibuprofen and codeine |
| Tramadol |
| Paracetamol and tramadol hydrochloride |
| Codeine |
| Paracetamol and dihydrocodeine tartrate |
| Triptans |
| Sumatriptan |
| Zolmitriptan |
| Rizatriptan |
| Ergot alkaloids |
| Nicergoline |
| Ergotamine caffeine |
| Dihydroergotoxine |
| Barbiturates |
| Antiemetics |
| Metoclopramide |
| Domperidone |
| Glucocorticoids |
| Mannitol injection |
| **Preventive medication** |
| Calcium antagonists |
| Flunarizine |
| Lomefloxacin |
| β1-receptor antagonists |
| Metoprolol |
| Bisoprolol |
| Propranolol |
| Atenolol |
| Antiepileptics |
| Magnesium valproate |
| Sodium valproate |
| Topiramate |
| Gabapentin |
| Antidepressants |
| Venlafaxine |
| Amitryptyline |
| Type A botulinum toxin |
| Others |
| Vitamin B_2_ |
| Coenzyme Q10 |
| Candesartan cilexetil |
| Prednisone |

NSAID: non-steroidal anti-inflammatory drug.
